# Mycolactone toxin induces an inflammatory response by targeting the IL-1β pathway: Mechanistic insight into Buruli ulcer pathophysiology

**DOI:** 10.1371/journal.ppat.1009107

**Published:** 2020-12-18

**Authors:** M. Foulon, M. Robbe-Saule, J. Manry, L. Esnault, Y. Boucaud, A. Alcaïs, M. Malloci, M. Fanton d’Andon, T. Beauvais, N. Labarriere, P. Jeannin, L. Abel, J. P. Saint-André, A. Croué, Y. Delneste, I. G. Boneca, L. Marsollier, E. Marion

**Affiliations:** 1 Université d’Angers, INSERM, CRCINA, Angers, France; 2 Laboratory of Human Genetics of Infectious Diseases, Necker Branch, INSERM, Paris, France; 3 Université de Paris, Imagine Institute, France; 4 Plateforme MicroPiCell, SFR santé François Bonamy, Nantes, France; 5 Institut Pasteur, Unité Biologie et Génétique de la Paroi Bactérienne, Paris, France; CNRS, INSERM, Équipe Avenir, Paris, France; 6 Université de Nantes, INSERM, CRCINA, Nantes; 7 Laboratoire d’Immunologie et Allergologie, CHU Angers, Angers, France; 8 Département de Pathologie Cellulaire et Tissulaire, CHU Angers, Angers, France; University of Massachusetts Medical School, UNITED STATES

## Abstract

Mycolactone, a lipid-like toxin, is the major virulence factor of *Mycobacterium ulcerans*, the etiological agent of Buruli ulcer. Its involvement in lesion development has been widely described in early stages of the disease, through its cytotoxic and immunosuppressive activities, but less is known about later stages. Here, we revisit the role of mycolactone in disease outcome and provide the first demonstration of the pro-inflammatory potential of this toxin. We found that the mycolactone-containing mycobacterial extracellular vesicles produced by *M*. *ulcerans* induced the production of IL-1β, a potent pro-inflammatory cytokine, in a TLR2-dependent manner, targeting NLRP3/1 inflammasomes. We show our data to be relevant in a physiological context. The *in vivo* injection of these mycolactone-containing vesicles induced a strong local inflammatory response and tissue damage, which were prevented by corticosteroids. Finally, several soluble pro-inflammatory factors, including IL-1β, were detected in infected tissues from mice and Buruli ulcer patients. Our results revisit Buruli ulcer pathophysiology by providing new insight, thus paving the way for the development of new therapeutic strategies taking the pro-inflammatory potential of mycolactone into account.

## Author summary

Buruli ulcer is a neglected tropical disease occurring mainly in poor rural areas of West and Central Africa. This cutaneous disease is caused by *Mycobacterium ulcerans*, a bacterium belonging to the same family as *M*. *tuberculosis* and *M*. *leprae*. The skin lesions are caused by a cytotoxic toxin named mycolactone, also known to act as an immunosuppressor and an anti-inflammatory molecule. However, Buruli ulcer lesions are characterized by a chronic cutaneous inflammation with a recruitment of cellular immune cells trying to counteract *M*. *ulcerans*. Our work allows for a reconcilitation of previous observations. We found by *in vitro* experiment on macrophages that the mycolactone-containing mycobacterial extracellular vesicles produced by *M*. *ulcerans* induced the production of IL-1β, a potent pro-inflammatory molecule, while other pro-inflammatory soluble factors are inhibited. We also detected IL-1β protein in a mouse model of *M*. *ulcerans* infection as well as in biopsies of Buruli ulcer patients. The pro-inflammatory potential of mycolacone has to be taken into account to understand the full pathophysiology of Buruli ulcer.

## Introduction

Buruli ulcer (BU), due to *Mycobacterium ulcerans* infection, is an emerging neglected tropical disease and the third most common mycobacterial disease worldwide, after tuberculosis and leprosy [1,2]. BU is considered to be a major health burden for affected populations (mostly children) and new areas of endemicity are emerging in both Africa and Australia [3,4]. This chronic infectious disease is characterized by massive skin tissue destruction associated with the main virulence factor of *M*. *ulcerans*, mycolactone, a lipid exotoxin [1]. Early and non-severe stages of BU can be treated with a two-month course of antibiotics [5,6]. At advanced stages, treatment requires a combination of surgery and antibiotic therapy at a major hospital. Patients rarely seek treatment before the disease reaches advanced stages, notably because the early stages are painless and afebrile [1]. Untreated lesions can spread to an entire limb and progress to chronic ulcers [7].

Considerable progress has been made toward understanding the early stages of the infection, especially through elucidation of the multifaceted roles of mycolactone in host colonization: (i) an immunomodulatory activity, targeting the Sec61 channel, allowing immune escape [8–11], (ii) an analgesic effect, targeting AT2R receptors, and accounting for the absence of pain in patients with early lesions [12,13] and, (iii) cytotoxic activity, potentially linked to the Sec61-mycolactone interaction, responsible for tissue destruction [8,14,15]. By contrast, much less is known about the mechanisms underlying disease progression at later stages, particularly the role of mycolactone during unabated inflammation.

### Mycolactone targeting the Sec61 channel: inhibition of cytokine secretion and activation of apoptosis pathways, key elements in early host colonization

Sites of *M*. *ulcerans* infection are surrounded by an immune infiltrate that is unable to control disease progression [16]. This lack of efficacy has been shown to result mostly from defective communication between immune cells. Indeed, mycolactone inhibits cytokine production by targeting Sec61 [8–11], a channel involved in the translocation of newly synthesized membrane and secreted proteins, including cytokines. Furthermore, interaction between mycolactone and Sec61 promotes cell death through an endoplasmic reticulum stress response, leading to apoptosis mediated by Bim [10,15]. These results were reinforced by a recent study showing a lower bacterial load and delayed lesion development in Bim-deficient mice [17]. Collectively, these findings highlight the importance of mycolactone-Sec61 interactions during host colonization occurring at the early stages of infection, but provide no information about their role at later stages.

### Late ulcerative and necrotizing stages are characterized by the activation of innate immune responses

After the pre-ulcerative phase, the ulcer grows rapidly, leading to the necrosis of subcutaneous tissue. Early stages are associated with an immunomodulatory environment required for bacterial colonization, as illustrated by the production of higher levels of the anti-inflammatory cytokine IL-10 in pre-ulcerative lesions than in ulcerative lesions [18,19], but *M*. *ulcerans* induces a strong inflammatory response at later stages. Indeed, although detected only indirectly, the local production of pro-inflammatory cytokines, such as IFNγ, in the infected tissues of BU patients appears to be greater at the ulcerative stage than at the pre-ulcerative stage [18,20,21]. Moreover, we have recently shown that spontaneous healing following prolonged ulceration is associated with a resolution of inflammation [22]. Finally, whereas the early stages of disease can mostly be attributed to the immunomodulatory activity of mycolactone, the advanced stages appear to be triggered by an exacerbation of the inflammatory response, promoting tissue necrosis. In this respect, the advanced stage of BU may therefore parallel the advanced stages of several other diseases, such as psoriasis, eczema, arthritis, mycobacterial and fungal infections [23–25].

In this context, we investigated the potential role of mycolactone in inducing a local pro-inflammatory response potentially contributing to the pathophysiology of Buruli ulcer. We observed that mycolactone, sequestered within extracellular vesicles (EVs) released by *M*. *ulcerans*, induced IL-1β production *in vitro*. We further dissected the underlying pathway and showed that (i) extracellular vesicles target TLR2 to generate the first signal required for pro-IL-1β production, (ii) mycolactone disturbs the cell membrane, inducing reactive oxygen species production (iii) triggering NLRP1 and NLRP3 inflammasome activation and the caspase-1 processing required for IL-1β production. We demonstrated that the injection of mycolactone-containing EVs elicited a strong local inflammatory response responsible for tissue damage, which could be prevented by corticosteroid treatment. An immune inflammatory signature was detected in BU patients, suggesting that, contrary to the current dogma, mycolactone may act as a pro-inflammatory toxin. We validated the relevance of this observation in the context of infection, by demonstrating the local production of inflammatory soluble mediators, including IL-1β, in the tissues of both infected mice and patients, confirming that Buruli ulcer causes an inflammatory response that may participate in tissue damage.

## Results

### Mycolactone promotes IL-1β secretion during *M*. *ulcerans* infection

**Fig 1:**
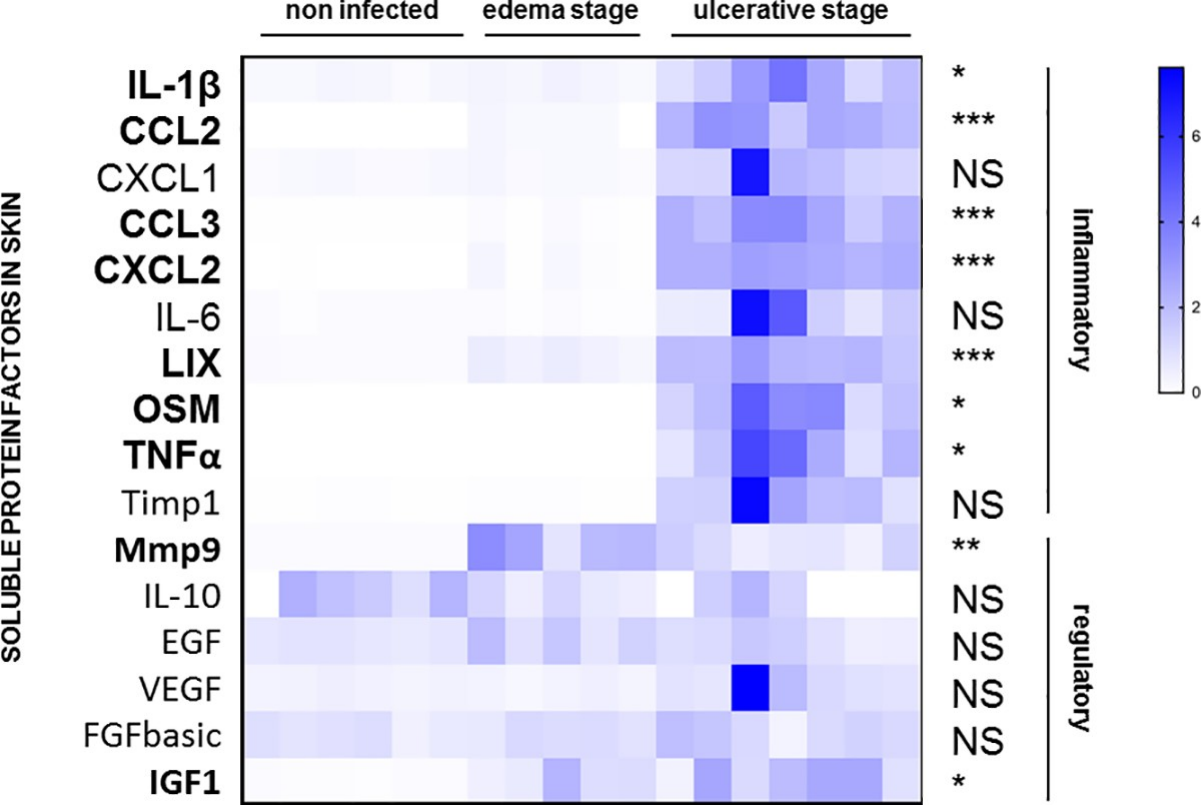
Pro-inflammatory soluble factors are locally overproduced during the ulcerative stage in a mouse model of *M*. *ulcerans* infection. Tail skin tissues were collected in non-infected mice and after subcutaneous inoculation of 1 × 10^4^ bacilli, at edema (D35) and ulcerative stages (D45). Skins were crushed and soluble protein factors were quantified by multiplex ELISA. A heatmap was used to represent the entire dataset. Benjamini, Krieger and Yekutieli *t-*tests with a false discovery rate (FDR) of 1% were performed for statistical analysis to compare non infected group and both edema or ulcerative groups. *P*-value: *p<0.5, **p<0.01, ***p<0.001. Each column corresponds to one mouse (*n* = 6 mice for non-infected stage, *n* = 5 for edema stage and *n* = 7 for ulcerative stage). IL, interleukin; CCL, chemokine ligand; CXCL, chemokine (C-X-C motif) ligand; LIX, LPS-induced CXC chemokine; OSM, oncostatin-M; TNFα, tumor necrosis factor alpha; TIMP, tissue inhibitor of metalloproteinases; MMP, matrix metalloproteinase; EGF, epidermal growth factor; VEGF, vascular endothelial growth factor; FGFbasic, basic fibroblast growth factor; IGF, insulin-like growth factor.

**Fig 2:**
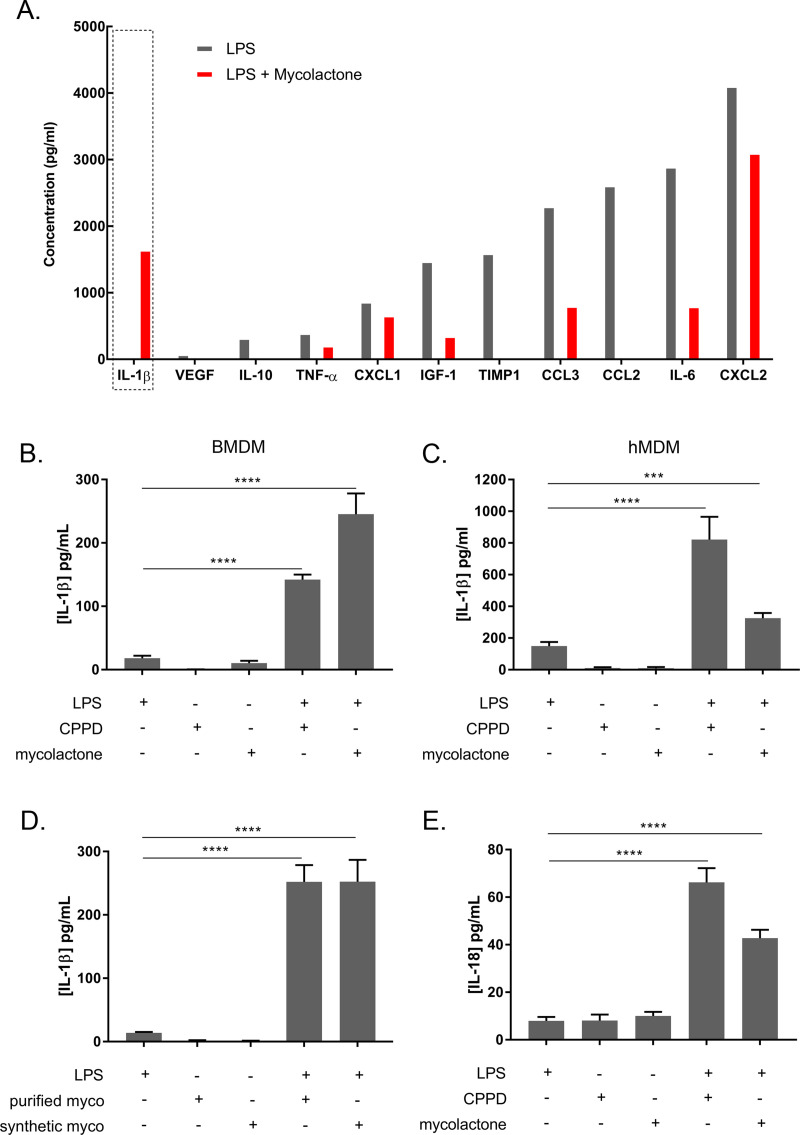
Mycolactone induces IL-1β secretion by LPS-stimulated human and mouse macrophages. **A.** Investigation of soluble factors in the supernatants of BMDMs after LPS stimulation (50 ng/mL) in the presence or absence of mycolactone (3 ng/mL). Soluble factors were quantified by multiplex ELISA. **B and C.** Mycolactone induced IL-1β secretion by bone marrow-derived macrophages (BMDMs) from C57Bl/6 mice (B) and human monocyte-derived macrophages (hMDMs) **D.** Synthetic mycolactone induced IL-1β production by bone marrow-derived macrophages (BMDMs) from C57Bl/6 mice. **E.** Mycolactone induced IL-18 secretion by bone marrow-derived macrophages (BMDMs) from C57Bl/6 mice. Cells were stimulated with LPS (50 ng/mL) and incubated with CPPD (20 μg/mL), or purified or synthetic mycolactone (3 ng/mL). Stimulations with LPS, CPPD or mycolactone alone were used as a control. IL-1β and IL-18 were quantified by ELISA in the 24 h cell culture supernatants. (**B-E**) Results are expressed as means + SEM of three independent experiments for BMDMs and 15 independent experiments for hMDMs. Nonparametric Mann-Whitney *U* tests (B to C and E) and Dunn’s multiple comparison tests (ANOVA) (D) were used for statistical analysis *P*-value. **p<0.01, ***p<0.001, ****p<0.0001.

We first re-evaluated the hypothesis that mycolactone may predominantly exert immunosuppressive effects during infection, particularly at the ulcerative stage. Using a proteomic approach (multiplex ELISA), we first quantified selected soluble factors in mouse tissues infected with *M*. *ulcerans* (Fig 1 and S1 Table). Seven of 10 pro-inflammatory cytokines tested were present in significantly larger amounts in ulcerative tissues than in non-infected skin tissues (IL-1β, CCL2, CCL3, CXCL2, LIX, OSM and TNFα). IGF-1 and MMP9 were the only regulatory mediators tested to display a significant increase in levels in ulcerative tissues. We then used a cellular assay to evaluate the impact of mycolactone on the secretion of immune mediators. Briefly, murine macrophages were stimulated with LPS to induce the secretion of soluble immune factors, and co-incubated with mycolactone or vehicle. We used a dose of 3 ng/mL mycolactone, as a higher dose (i.e. 30 ng/ml) known to inhibit the secretion of IL-6 or TNFα fully resulted in a significant increase in cytotoxicity (assessed by bioluminescent cytotoxicity assay) (S1A and S1B Fig) and cellular apoptosis (assessed by TUNEL assay) (S1C, S1D and S1E Fig). In agreement with previous studies [26,27], mycolactone decreased the production of several soluble mediators, including both inflammatory and regulatory cytokines (Fig 2A). Surprisingly, mycolactone greatly increased the production of IL-1β by LPS-activated macrophages. We then confirmed that mycolactone induced IL-1β production in murine macrophages, in a similar manner to calcium pyrophosphate dihydrate crystals (CPPD) (Fig 2B), used as a positive control for IL-1β induction [28]. We observed a similar effect on human macrophages (Fig 2C). Similar results were also obtained with synthetic mycolactone, excluding effects of potential contaminants present in purified mycolactone (Fig 2D). As IL-18 is closely related to IL-1β (same unconventional secretion mechanism and similar pro-inflammatory properties), we also investigated the impact of mycolactone on IL-18 secretion. As shown in Fig 2E, mycolactone also slightly but significantly induced IL-18 secretion by LPS-activated murine macrophages. Finally, no IL-1β was produced in the absence of LPS, demonstrating that mycolactone alone is not sufficient to induce IL-1β secretion. Our results therefore suggest that mycolactone may be involved in the initiation of inflammatory responses through its ability to induce the production of IL-1β, which is known to play a key role in such phenomena [29].

### Transcriptomics on whole blood from a BU patient identifies a signature of inflammation

**Fig 3:**
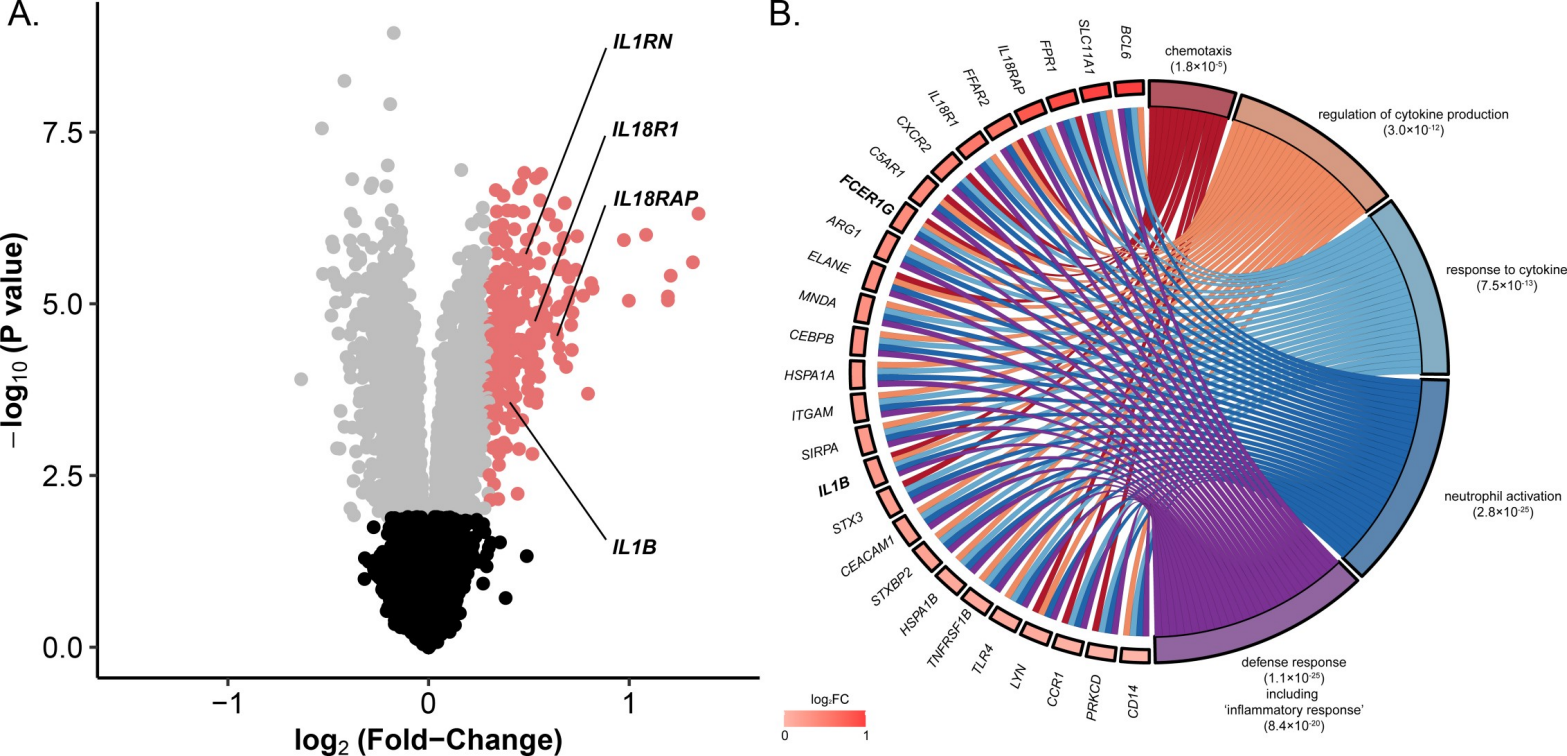
Inflammatory response signature of circulating whole-blood cells from patients with PCR-confirmed active Buruli ulcer. Identification and functional annotation of genes overexpressed in BU patients at diagnosis relative to the end of treatment. (A) Volcano plot of the 11,884 genes tested for differential expression between diagnosis and the end of treatment. Black dots correspond to genes that are not differentially expressed, gray dots correspond to differentially expressed genes with a log_2_(FC) < 0.3, and red dots correspond to genes overexpressed at diagnosis (log_2_(FC) > 0.3). IL1 family genes that are significantly differentially expressed are labeled. (B) Gene ontology enrichment analysis for genes upregulated at diagnosis. Chord diagram showing the 27 genes (left) tagged by at least four of the five GO groups (right) identified with ClueGO (see Materials and Methods). Rectangles following gene symbols indicate the log_2_(FC). GO group adjusted *P*-values (in parentheses) were calculated from the difference between the observed and expected numbers of upregulated genes at diagnosis annotated with a given GO group among the genes for which expression was successfully measured. The “inflammatory response” GO term, which belongs to the “defense response” GO group, and its adjusted *P*-value are displayed. All GO terms for which significant enrichment was detected are shown in S2 Table.

We then used a transcriptomic approach to provide further evidence for the existence of an inflammatory signature in *M*. *ulcerans*-infected patients. We analysed the cytokine profile of circulating immune cells isolated from 29 PCR-confirmed Buruli ulcer patients. In whole-blood cells from these patients, 3,020 genes displayed significant differential expression between the start and end of treatment. However, as anticipated on the basis of the presumed local host response to *M*. *ulcerans*, only 241 of these genes had a log2(FC) > 0.3 and an FDR *q*-value < 0.05 (Fig 3A, S2 Table). Gene ontology (GO) enrichment analysis for the 241 genes more strongly expressed at diagnosis than at the end of treatment identified 46 GO terms with an FDR *q*-value < 0.01 (S3 Table). The genes were each tagged by several GO terms, and the 46 GO terms were clustered into five GO groups (see Materials and Methods) (Fig 3B). In total, 28 GO terms clustered in the “defense response” group (GOGroup corrected *p*-value = 1.1x10^-25^) (Fig 3B). Within this group, the GO term “inflammatory response” (fold-change enrichment of 4.3; FDR *q*-value = 8.4x10^-20^) was captured (Fig 3B). Genes tagged by this GO term included *IL1B*, *IL10R1*, *IL10RB*, *IL18R1*, *IL18RAP*, *SCL11A1*, *TLR4*, *TLR5*, *CCR1*, *CXCR2* and *NLRC4* (S3 Table). Importantly, we identified a core gene set of 27 genes belonging to at least four GO groups (Fig 3B). Only *IL1B* and *FCER1G* were present in the five GO groups, identifying these genes as drivers of our GO analysis. In addition, 20 of the genes from the core gene set were tagged with the “inflammatory response” term. Taken together, these results made it possible to identify genes involved in inflammation that were more strongly expressed at diagnosis than at the end of treatment, revealing the key role of inflammation, and probably of *IL1B*, in BU pathogenesis.

### Deciphering the pathways involved in mycolactone-induced IL-1β production

The secretion of IL-1β requires two signals: (i) the first signal, which can be supplied by LPS for example, triggers the production of pro-IL-1β and (ii) the second signal leads to the activation of caspase-1 (downstream inflammasome), which is required for the processing of pro-IL-1β and the secretion of mature IL-1β (29). We found that LPS was required to demonstrate the role of mycolactone in the induction of IL-1β (Fig 2). Cells incubated with mycolactone alone did not produce IL-1β, or even pro-IL-1β (S1F Fig). These findings indicate that mycolactone supplies the second signal. In this context, we deciphered the activation pathway involved in the mycolactone-induced IL-1β secretion in mouse macrophages. For this, mycolactone was compared to CPPD, used as an inducer of the conventional IL-1β maturation pathway [28].

#### (i) Role of reactive oxygen species and membrane disturbance

**Fig 4:**
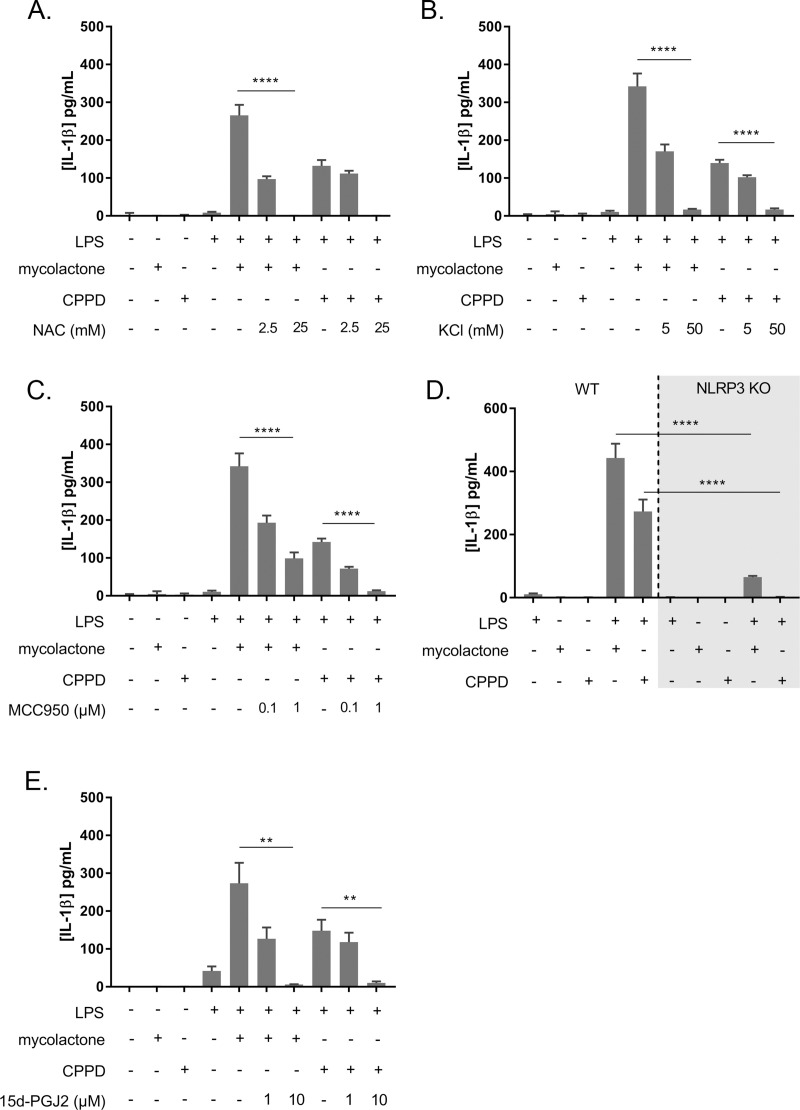
Mycolactone induces IL-1β secretion by activating the NLRP3/1 inflammasome pathway. Three known inhibitors of the inflammasome pathway implicated in the secretion of IL-1β were added to LPS-stimulated BMDM cultures in the presence of mycolactone (3 ng/mL) or CPPD (20 μg/mL). **A.** The addition of 2.5 mM or 25 mM N-acetyl-L-cysteine (NAC) decreased and totally abolished IL-1β secretion, respectively, in the presence of either mycolactone or CPPD. **B.** The addition of 5 mM or 50 mM KCl to the medium decreased and abolished IL-1β secretion, respectively, in the presence of either mycolactone or CPPD. **C.** The addition of MCC950, a specific inhibitor of the NLRP3 inflammasome, at a concentration of 0.1 μM or 1 μM, significantly decreased IL-1β secretion in the presence of either mycolactone or CPPD. **D.** IL-1β secretion levels were significantly lower in LPS-stimulated BMDMs with a knockout (KO) for NLRP3, in the presence of either mycolactone (3 ng/mL) or CPPD (20 μg/mL), than in wild-type BMDMs. **E.** The addition of 15-deoxyΔ12,14-prostaglandin J2, which inhibits both the NLRP3 and NLRP1 inflammasomes, at a concentration of 10 μM or 1 μM, decreased and abolished IL-1β secretion, respectively, in the presence of either mycolactone or CPPD. Results are expressed as means + SEM of three independent experiments (triplicates of each condition in each independent experiment). Nonparametric Mann-Whitney *U* tests were used for the statistical analysis. *P*-value: *p<0.5, **p<0.01, ***p<0.001, ****p<0.0001.

Reactive oxygen species (ROS) are known to induce caspase-1 recruitment by activating the inflammasome [29]. Interestingly, mycolactone has been shown to induce ROS production in keratinocytes and macrophages [30,31]. We used N-acetyl-L-cysteine (NAC), a potent inhibitor of ROS, to evaluate their role in mycolactone-induced IL-1β production. NAC abolished the induction of IL-1β production induced by mycolactone or CPPD (Fig 4A), demonstrating the involvement of ROS in this process. Cell membrane disturbance, leading to potassium efflux, leads to ROS production. Mycolactone has been reported to disturb the cell membrane [32,33] and cause potassium efflux [12]. We investigated the impact of the cell membrane modifications induced by mycolactone on IL-1β production. Treatment with KCl, which reduces potassium efflux, inhibited the induction of IL-1β production by mycolactone (Fig 4B). A similar inhibition was observed with CPPD, which also disrupts the membrane lipid bilayer [34]. This result suggests that mycolactone can induce IL-1β production, probably through membrane disruption and ROS production similar to that induced by CPPD.

#### (ii) Role of NLRP3 and NLRP1 in the IL-1β secretion induced by mycolactone

NLRP3 is the main inflammasome complex triggered in response to bacterial infections or toxins inducing membrane disturbances, such as mycolactone [32,35]. We used MCC950, a specific inhibitor of the NLRP3 inflammasome, to evaluate its role in the IL-1β secretion induced by mycolactone. MCC950 inhibited 71% (maximum effect) of the IL-1β production induced by mycolactone, and 100% of that induced by CPPD (Fig 4C). In parallel, we evaluated the IL-1β production induced by mycolactone in NLRP3^-/-^ macrophages (Fig 4D). IL-1β production was only partially decreased in response to mycolactone (85% inhibition relative to macrophages derived from WT mice) and was completely abolished with CPPD. Together, these results suggest that mycolactone induces IL-1β production partly by targeting the NLRP3 inflammasome. NLRP1, another major inflammasome, is also known to induce IL-1β secretion [29]. We therefore used 15-deoxyΔ12,14-prostaglandin J2 (15d-PGJ2) to inhibit both the NLRP3 and NLRP1 inflammasomes [35]. We found that 10 μM 15d-PGJ2 abolished the production of IL-1β induced both by CPPD and mycolactone (Fig 4E). Thus, mycolactone mostly targets NRLP3, but also NLRP1 inflammasomes, to induce the production of IL-1β by macrophages.

### Mycobacterial EVs containing mycolactone trigger IL-1β production by macrophages through TLR2

**Fig 5:**
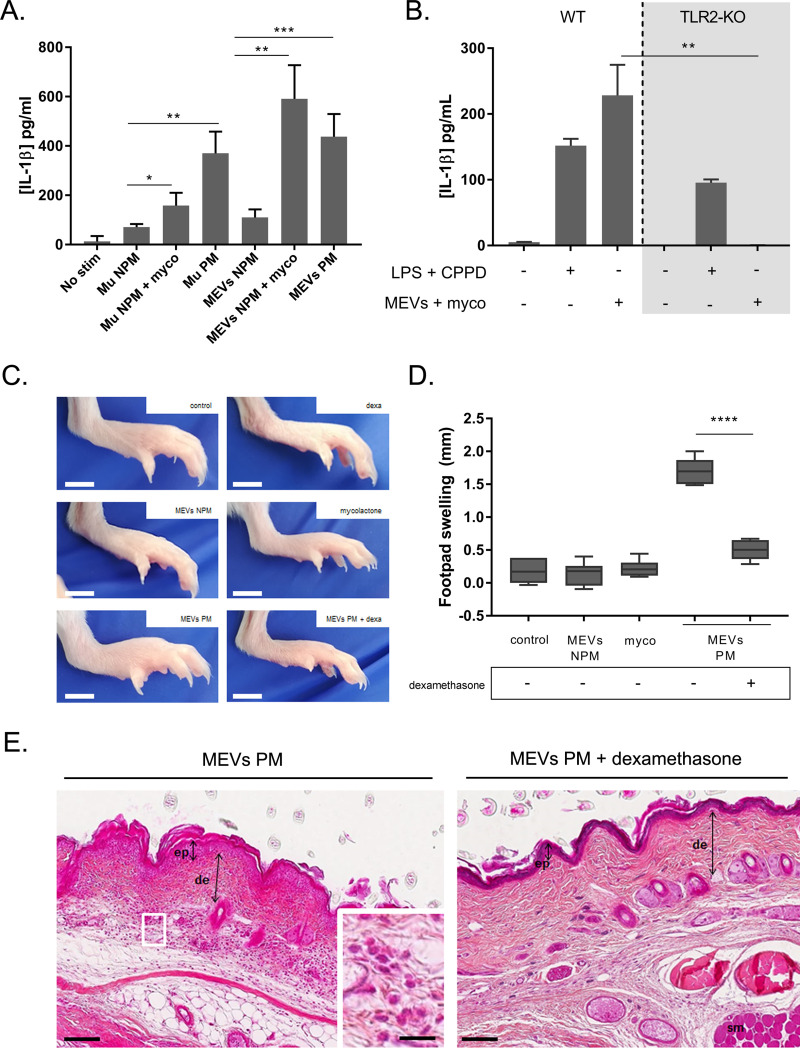
Mycobacterial extracellular vesicles containing mycolactone induce IL-1β *in vitro* and an inflammation *in vivo* which is prevented by corticosteroids. **A.** *M*. *ulcerans* bacteria or *M*. *ulcerans* extracellular vesicles (MEVs) induced IL-1β secretion by human monocyte-derived macrophages (hMDMs). Cells were stimulated with vesicles (1x10^8^) from an *M*. *ulcerans* strain unable to produce mycolactone (MEVs NPM) or with bacteria of this strain (Mu NPM) (MOI 50), and incubated with mycolactone (3 ng/mL). In parallel, cells were stimulated with vesicles (2.5x10^8^) from an *M*. *ulcerans* strain producing mycolactone (MEVs PM) or with bacteria of this strain (Mu PM) (MOI 50). Stimulations with MEVs NPM or Mu NPM alone were used as controls. IL-1β was quantified by ELISA in the 24 h cell culture supernatants. Results are expressed as the means + SEM of eight independent experiments. **B.** MEVs induced IL-1β secretion by bone marrow-derived macrophages (BMDMs) through Toll-like receptor 2 (TLR2). Wild-type and TLR2-KO cells were stimulated with LPS or with MEVs NPM and incubated with CPPD (20 ng/mL) or with mycolactone (3 ng/mL). IL-1β was quantified by ELISA in the 24 h cell culture supernatants. Results are expressed as the means + SEM of two independent experiments, Dunn’s multiple comparison tests (ANOVA) were used for statistical analysis *P*-value: **p<0.01. **C.** BALB/c mice received a single dose of vehicle, purified mycolactone alone (2 μg; myco), MEVs containing mycolactone (2 μg; MEVs PM) or MEVs purified from NPM strain (at equivalent particles quantity to MEVs PM; MEVs NPM) in the footpad. Dexamethasone treated groups received dexamethasone at 10 mg/kg by oral gavage for three consecutive days before the subcutaneous injections. Footpads were photographed 24 h after injections. **D.** Footpad welling was measured before and 24 h after the injections. Footpad swelling (*value at 24 h post-injection–value before injection*) was significantly increased after injection of MEVs containing mycolactone; this effect was prevented by dexamethasone treatment (6–9 mice/group). Results are expressed as box-and-whisker plots, from the minimum to the maximum value. Tukey’s multiple comparison test (ANOVA) was used for statistical analysis; ***p<0.0001. **E.** HPS staining evidence an inflammatory cell infiltrate after the injection of MEVs containing mycolactone into untreated mice (left), but not in mice treated with dexamethasone (right). ep, epidermis; de, dermis; sm, striated muscle. Scale bars: 500 μm (inset: 50 μm).

Having demonstrated that mycolactone can provide the second signal required for IL-1β production, we next evaluated the nature of the first signal in the context of *M*. *ulcerans* infection. The bacterial endotoxin LPS has emerged as a major first signal in several types of bacterial infections [29,36], but not in mycobacterial infections. It was thus crucial to demonstrate that whole bacillus or a mycobacterial component could provide the first signal required for IL-1β synthesis. We found that the incubation of human macrophages with a *M*. *ulcerans* (whole bacillus) strain producing mycolactone (Mu PM) induced IL-1β production at levels similar to that induced by LPS and mycolactone (Fig 5A), whereas no IL-1β was produced with a *M*. *ulcerans* strain defective for mycolactone production (Mu NPM) (Fig 5A). The incubation of human macrophages with mycolactone and Mu NPM bacilli also induced the secretion of IL-1β (Fig 5A). These results demonstrate that mycobacterial components provide the first signal required for IL-1β production, and confirm the role of mycolactone as the second signal.

We previously demonstrated that mycobacterial species, especially *M*. *ulcerans*, can secrete extracellular vesicles derived from the bacterial membrane (MEVs) and carrying mycolactone, which are detected even in the systemic circulation in patients with active Buruli ulcer [37]. Here, we hypothesized that vesicles containing mycolactone would be able to induce IL-1β production, thus providing both the first and second signals. We showed that MEVs purified from a Mu PM strain induced IL-1β production in human macrophages (Fig 5A), whereas vesicles devoid of mycolactone (purified from a Mu NPM strain) did not. IL-1β production was restored by co-incubating the vesicles isolated from the NPM strain with mycolactone. These results underline demonstrate that MEVs carrying mycolactone efficiently induce IL-1β production.

The first signal for IL-1β production requires Toll-like receptor (TLR) stimulation, and LPS has already been reported to provide this signal via TLR4 [38]. Mycobacterial membrane components, including MEVs, are known to be activated via TLR2 [39]. In this context, we investigated IL-1β production by TLR2-deficient (TLR2^-/-^) BMDMs. Cells stimulated with MEVs from the NPM strain (first signal), and incubated with mycolactone (second signal), produced IL-1β only in the presence of TLR2, this production being abolished in TLR2^-/-^ cells (Fig 5B). Stimulation with LPS and incubation with CPPD led to IL-1β production regardless of the presence or absence of TLR2. Thus, MEVs provide the first signal required for IL-1β production, in a TLR2-dependent manner.

### MEVs containing mycolactone induce a strong inflammatory response *in vivo* that is prevented by corticosteroids

Previous studies aiming to decipher the role of mycolactone *in vivo* showed that the toxin was involved in tissue destruction at high doses [40]. Here, we aimed to re-evaluate the potential of mycolactone to induce an inflammatory response in a more pathophysiological context. To this end, MEVs containing mycolactone were injected into the footpads of mice in the presence or absence of the anti-inflammatory drug dexamethasone, and the effect of mycolactone was assessed by monitoring tissue swelling and performing histological analyses at different time points. We found that 2 μg of mycolactone in MEVs induced footpad swelling 24 h after inoculation (Fig 5C), and that dexamethasone administration entirely prevented this swelling (Fig 5D). No such swelling was induced by MEVs purified from an *M*. *ulcerans* strain unable to produce mycolactone (NPM), or by 2 μg of purified mycolactone. Histological analysis revealed that the swelling was accompanied by an edema with an inflammatory cellular infiltrate consisting mostly of mononuclear cells localized in the dermis (Fig 5E). As 15d-PGJ2 has showed some anti-inflammatory properties in previous *in vivo* studies [41,42], and fully inhibited IL-1β secretion in our previous *in vitro* experiments (Fig 4E), we administrated it in mice injected with MEVs containing 2 μg mycolactone. Footpad swelling of mice treated (every 3 hours) with 15d-PGJ2 was significantly lower in comparison to untreated mice (S2A Fig, Bonferroni’s multiple comparison test; * p-value<0.05, ** p-value<0.005). This decrease was less pronounced than the decrease observed in mice treated with dexamethasone (Fig 5D), which can be explained in part to the poor stability of 15d-PGJ2 in the mice organism after injection [43] or the involvement of non-inflammasome mediators. Finally, a higher dose of mycolactone (12 μg) provoked tissue destruction, which was also prevented by dexamethasone adminstration (S2B Fig). Thus, the mycolactone present in MEVs triggered a strong inflammatory response that was involved in tissue damage.

### IL-1β is detected at distance from the infectious core in Buruli ulcer patients

**Fig 6:**
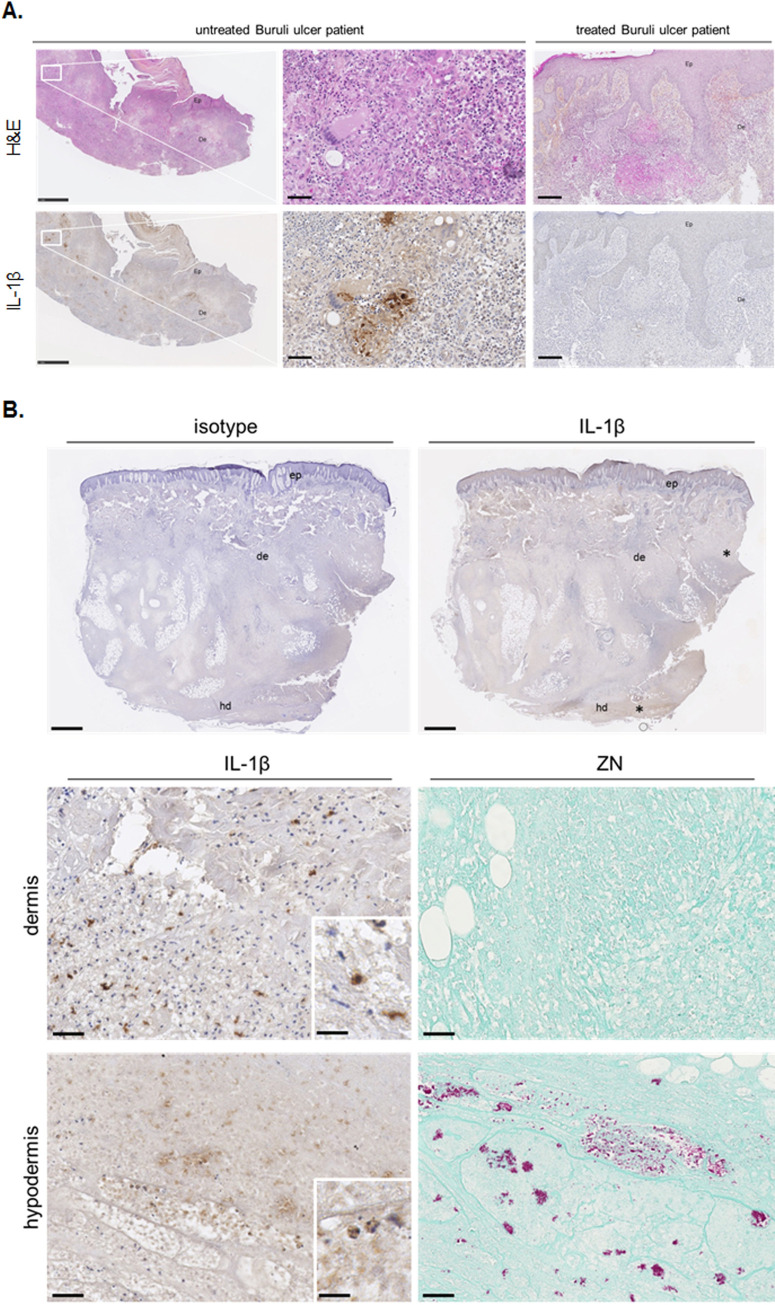
IL-1β is produced in the pathophysiological context of *M*. *ulcerans* infection. **A.** IL-1β was detected by immunohistochemistry in the lesions (category II according to WHO, ulcerative stage) of patients with active Buruli ulcers (untreated) but not in the lesions of patients with treated Buruli ulcers (antibiotic treatment) (here are representative images of *n* = 2 for active Buruli ulcer lesions, *n* = 6 for treated Buruli ulcer lesions). The inset shows specific staining for IL-1β in clusters of histiocytes. Scale bars: 250 μm (inset = 50 μm). **B.** Detection of IL-1β at some distance from the acid-fast bacilli (AFB) detected in the hypodermis by Ziehl-Neelsen staining (right panel). Scale bars: 1 mm for the upper panel and 50 μm for the lower panel (inset = 20 μm).

Finally, to demonstrate the secretion of IL-1β in the context of Buruli ulcer, we performed histological analyses on biopsy specimens from patients with PCR-confirmed disease. IL-1β was detected in active *M*. *ulcerans* lesions: numerous clusters of histiocytes and areas of inflammation were stained for IL-1β (Fig 6A). Interestingly, IL-1β staining was observed in the dermis, at some distance from the staining for acid-fast bacilli (AFB), which were found only in the hypodermis (Fig 6B). No IL-1β was detected in the lesions of treated patients, despite the presence of a large amount of inflammatory infiltrate (Fig 6A). These results demonstrate that IL-1β is produced in active Buruli ulcer lesions. With the same approach, we also detected IL-12, another pro-inflammatory cytokine, which was produced in larger amounts in active Buruli ulcer lesions than in lesions from treated patients (S3A and S3B Fig). These results, together with systemic inflammatory and bacterial infection signatures, suggest that the induction of IL-1β production by the mycobacterium and its toxin may contribute to the initiation of uncontrolled local inflammation, inducing tissue damage. Finally, the detection of IL-1β at some distance from the bacilli may reflect the diffusion of mycolactone via the MEVs.

## Discussion

### Mycolactone displays a pro-inflammatory activity leading to IL-1β production

Mycolactone was thought to shut down immune responses by interacting with Sec61, thereby preventing protein processing in the ER [8–11]. Interestingly, one of the first studies dissecting this mechanism showed that, the production of IL-1β, unlike that of other cytokines, was only partially blocked by mycolactone in human monocytes (depending on the TLR ligand used to activate cells) [27]. The authors suggested at the time that mycolactone did not inhibit inflammasome activity, but it has never before been suggested that mycolactone might instead, in some cases, induce this pathway. We dissected the pathway targeted by *M*. *ulcerans* and its mycolactone toxin in the induction of a pro-inflammatory response. We reveal here that mycolactone induces an inflammatory response involved in tissue damage. We found that, in the context of Buruli ulcer, the mycolactone produced by *M*. *ulcerans* or present in extracellular vesicles derived from this bacillus, induces IL-1β secretion by macrophages. IL-1β production involves two steps, controlled by different stimuli. We first demonstrated that the bacillus, and its extracellular vesicles (known to contain TLR2 ligands, such as mycobacterial lipoproteins, lipopeptides and lipids [39]) promoted the production of pro-IL-1β, as already reported for LPS. Thus, mycolactone, probably through membrane disturbance and the ROS production it triggers, induces NLRP3/1 inflammasome activation, leading to the release of active IL-1β.

### *M*. *ulcerans* and mycolactone promote a pro-inflammatory environment, providing new insight into the pathophysiology of Buruli ulcer and the properties of mycolactone

IL-1β is a potent inflammatory cytokine that plays a key role in inflammatory responses by directly or indirectly activating several immune cellular actors and inducing soluble immune factors [29]. Thus, mycolactone-induced IL-1β release may play a role at different stages of *M*. *ulcerans* infection. For example, mycolactone has been shown to induce Bim-dependent apoptosis during early host colonization [17,44]. Apoptosis is a mechanism known to be involved in IL-1β secretion [45], but also to be induced by IL-1β [46]. We cannot, therefore, exclude the possibility that mycolactone-induced IL-1β secretion is at least partly responsible for cell apoptosis. IL-1β is also known to promote B-cell activation and antibody production [47–49]. We previously showed that the humoral response and antibody production are stronger at later stages of *M*. *ulcerans* infection [50]. In particular, we have shown that, at the ulcerative stage, there is a local accumulation of IgM, highlighting the pro-inflammatory environment, as already reported in other inflammatory situations in the skin [51]. We also detected the local presence of IL-12, another pro-inflammatory cytokine for which production depends on IL-1β and IFN-γ secretion [52]. The local production of IFN-γ has already been reported to occur in patient tissues at later stages of infection [18,20,21]. We also observed an exacerbation of the pro-inflammatory response, even at systemic level, at later stages of infection. Moreover, we and others [16,17] have demonstrated the occurrence of an inflammatory response at some distance from the infection site, consistent with the presence of diffusing pro-inflammatory mediators. We show here that MEVs carrying mycolactone induce IL-1β secretion, accounting for the inflammatory response observed some distance away from the infection site, as already reported for other mycobacteria [39].

Our data therefore show that *M*. *ulcerans* and its toxin create a pro-inflammatory environment, disrupting tissue homeostasis and resulting in tissue damage. A sustained pro-inflammatory response is known to be involved in immune disorders associated with skin damage, such as lupus or ulcerative colitis, for example [53]. In the context of infection, it has been shown that chronic infections with certain microorganisms causing prolonged inflammation, such as *Helicobacter pylori* in the stomach or *Mycobacterium marinum* in the skin, may be responsible for continual damage to the tissues, potentially even leading to tumor initiation in the long term [54,55]. The development of squamous cell carcinoma has already been described in several BU cases, after a prolonged ulcerative phase [56,57].

### Mycolactone probably impacts other pro-inflammatory cytokines in addition to IL-1β

This transcriptomic study of whole-blood cells from BU patients is the first attempt to determine which genes are differentially expressed during BU pathogenesis. One strength of this study is that it was performed *in natura*. Indeed, gene expression levels were quantified at BU diagnosis and after the completion of treatment. Whole-blood cells were not stimulated *in vitro*. The diagnosis time point thus represents a good proxy for an active BU episode, whereas the end of treatment time point should be a good proxy for the return to the basal state. In other words, genes more strongly expressed at diagnosis than at the end of treatment are probably induced by *M*. *ulcerans* and mycolactone. The 241 genes upregulated at diagnosis, including the 27 genes of the core gene set in GO analysis in particular, thus represent good candidates for involvement in BU pathogenesis.

We have shown that IL-1β plays a key role in BU pathogenesis. However, other inflammatory pathways may also be involved. For example, our transcriptomic approach made it possible to quantify the expression of 10 genes of the IL1 family, including genes encoding four ligands (*IL1B*, *IL1RN*, *IL18*, *IL36B*), three receptors (*IL1R2*, *IL1RL1*, *IL18R1*), and three coreceptors (*IL1RAP*, *IL18RAP*, *SIGIRR*). Four (*IL1B*, *IL1RN*, *IL18R1* and *IL18RAP*) of these 10 genes were significantly more strongly expressed at diagnosis than at the end of treatment (Fig 3A). IL1B, IL18R1 and IL18RAP encode proteins having pro-inflammatory properties, whereas IL1RN is a known antagonist of IL1A and IL1B [58]. It remains unclear whether IL-1RN counteracts IL-1β in the whole-blood cells of BU patients. Strikingly, the *IL18R1* and *IL18RAP* genes from the IL-18 pathway were more strongly expressed at diagnosis, suggesting that inflammation may be triggered by IL-18 in addition to IL-1β, a hypothesis supported by the ability of mycolactone to induce the secretion of this cytokine.

Overall, our findings indicate that *M*. *ulcerans* and its toxin may provoke immune disorders, consecutive to an uncontrolled pro-inflammatory response that enhances tissue damage at later stages of infection. Our results demonstrate that mycolactone is more than just a destructive and immunosuppressive toxin, and highlight the “Machiavellian” strategy used by *M*. *ulcerans* to colonize its host through the synthesis of a toxin with pleiotropic effects.

In conclusion, we have demonstrated that the infiltrate of innate and adaptive immune cells present at the site of infection is not entirely incapacitated by mycolactone. Instead, this toxin promotes a pro-inflammatory environment, leading to tissue destruction and/or progression of the infection. Finally, the whole range of activities of mycolactone (whether in vesicles or bacilli), from cytotoxic effects involving immunosuppression to the induction of inflammation, may be observed progressively in cutaneous lesions. These activities lead to a failure of the immune system to neutralize *M*. *ulcerans* and to control the infection process. Efforts could now be made to develop a therapeutic tool targeting the effects of mycolactone, taking the pro-inflammatory action of this toxin into account.

## Materials and methods

### Ethics statement for animal experiments and the use of human tissues

All animal experiments were performed in accordance with national (articles R214-87 to R214-90 of the French “rural code”) and European (directive 2010/63/EU of the European Parliament and of the Council of September 22, 2010 on the protection of animals used for scientific purposes) guidelines. All protocols were approved by the ethics committee of the Pays de la Loire region, under protocol no. APAFIS8904. Mice were housed in specific pathogen-free conditions in the animal house of Angers University Hospital, France (agreement A 49 007 002). The use of biopsy samples from patients for research purposes was approved by the research committee of the government of Benin (Ministry of Health, Republic of Benin, agreement number 2893). The genetic studies of susceptibility to Buruli ulcer (BU) were approved by the institutional review board of the CDTLUB (*Centre de Diagnostic et de Traitement de la Lèpre et de l’Ulcère de Buruli*) and the national Buruli ulcer control authorities in Benin (IRB00006860), and by the ethics committee of Angers University Hospital, France. All participants provided written informed consent or had their parents provide written informed consent on their behalf.

### *M*. *ulcerans*, vesicles and mycolactone

*Mycobacterium ulcerans* strains 01G897 and 1615 were originally isolated from patients from French Guiana and Malaysia, respectively [14,59]. *M*. *ulcerans* 01G897 was used for animal inoculation, while *M*. *ulcerans* 1615 and its mycolactone-deficient mutant (Tn:118 mutant, [60]) were used for mycolactone and/or MEV purification. These strains were grown on solid 7H10 medium supplemented with 10% OADC, as previously described [37]. After 35 days of culture, mycolactone or vesicles were purified from the pellet, as previously described [37,61]. Mycolactone was stored in absolute ethanol at -20°C in the dark, and vesicles were stored in PBS at -80°C.

### Vesicle characterization: size and concentration

*M*. *ulcerans* vesicles were characterized by nanoparticle tracking analysis (NTA) on the Nanosight NS300 system (Malvern Instruments Ltd., Malvern, United Kingdom) equipped with a sCMOS camera and a blue laser (488 nm) to illuminate particles within the size range of 10–2000 nm. The sample was loaded into the analysis chamber with a syringe pump at a constant flow rate. Nanoparticles were illuminated by the laser and their movement under Brownian motion was tracked for 60 s at camera level 13. Five videos were captured to provide significant concentration and size data. During the analysis, viscosity was set to that of water and the detection threshold was set at a pixel value of 5. All videos were subjected to NTA with Nanosight particle tracking software to provide nanoparticle concentration and size distribution profiles (S4 Fig). The software tracks many particles individually and the Stokes-Einstein equation is used to calculate their hydrodynamic diameters.

### Mycolactone concentration

The quantity of purified mycolactone, or mycolactone present in the MEVs, was evaluated by high performance liquid chromatography (HPLC) on a C18 column. Mycolactone was eluted with a 33-min gradient from 90% to 10% water for phase A and 10 to 90% acetonitrile for phase B, with a flow rate of 1 mL/min. The chromatogram was monitored at 363 nm, logε = 4.29, and the mycolactone peak was quantified by determining the area under the curve.

### Murine bone marrow-derived macrophages

Bone marrow-derived macrophages (BMDMs) were generated by culturing bone marrow progenitors (5 x 10^5^ cells/mL) from the femur bones of wild-type and knockout C57Bl/6 mice for seven days in the presence of M-CSF (20 ng/mL). Macrophages were used to seed 96-well plates at a density of 10^5^ cells/well.

### Human peripheral blood mononuclear cells

Peripheral blood mononuclear cells (PBMCs) were obtained from healthy human volunteers (from the blood collection center, Angers, France; agreement ANG-2003-2). CD14^+^ monocytes were isolated by magnetic sorting and macrophages were generated by culturing monocytes for five days with 50 ng/mL GM-CSF. Differentiated macrophages were used to seed 96-well plates at a density of 10^5^ cells/well.

### Cellular tests on BMDMs and PBMCs

We added CPPD (InvivoGen), mycolactone, *M*. *ulcerans* or vesicles, at various concentrations, with or without KCl (Sigma P5405) to inhibit K^+^ channels; MCC950 (InvivoGen inh_mcc), an NLRP3 inhibitor; 15-deoxyΔ12,14-prostaglandin J2 (Cayman Chemicals ref. 18570), an NLRP3/1 inhibitor; N-acetyl-L-cysteine (NAC, Sigma A9165), a ROS inhibitor; and Z-VAD-FMK (InvivoGen tlrl_vad), a pan-caspase inhibitor. The macrophages were then incubated for 24 h with or without LPS (from *E*. *coli* serotype O111:B4; Sigma-Aldrich) added to a final concentration of 50 ng/mL.

### Animal procedures: infection with *M*. *ulcerans*, inoculation with mycolactone, dexamethasone

A bacterial suspension of *M*. *ulcerans* 01G897 was prepared, as previously described [22,37] and its concentration was adjusted to 2 x 10^5^ acid-fast bacilli/mL for an inoculum of 1 x 10^4^ bacilli in 50 μL, which was injected into the tail dermis of six-week-old female consanguineous C57Bl/6 mice (Janvier, Le Genest Saint Isle, France). For footpad swelling assays, MEVs diluted in PBS or mycolactone diluted in corn oil supplemented with 8% ethanol (as previously described [62]) were subcutaneously injected, in a volume of 15 μL, into the footpad of seven-week-old female BALB/c mice (Charles River Laboratories, Saint-Germain-Nuelles, France). For groups treated with dexamethasone, mice received 10 mg/kg orally in 100 μL, once daily for three days before mycolactone injection. For groups treated with 15d-PGJ2, mice received 1 mg/kg by intraperitoneal route in 100 μL, every three hours during 12 hours after MEVs injections.

### Histology

After the inoculated mice had been killed, the footpad was excised and immediately fixed by incubation in 4% paraformaldehyde (PFA) for 24 h. Skin biopsy specimens were provided by the CDTLUB of Pobé (Benin) and stored in 4% PFA. Tissues were then embedded in paraffin blocks, which were cut into 3 μm-thick sections. Hematoxylin-phloxine-saffron (HPS) staining was performed, according to the manufacturer’s protocol. For immunohistochemical staining, the sections were subjected to pretreatment involving antigen retrieval by heating in sodium citrate buffer for 20 mins. The samples were then incubated with a rabbit polyclonal anti-IL1β antibody (Abcam ref. #ab9722), a rabbit anti-IL-12 antibody (Bioss, ref. #bs-1789R), both at a dilution of 1:300, or a rabbit IgG (Vector Laboratories, ref. #I-1000) as isotype control, at room temperature for 1 hour, and then with a conjugated secondary antibody. They were then stained with DAB.

### Extraction of proteins from mouse tails

Infected mice were killed at various time points after infection. Skin samples were excised and ground with a TissueRuptor, in PBS containing Complete EDTA-free cocktail. The resulting suspensions were treated with 30 μg/mL DNAse and 5 mM CaCl_2_ and centrifuged at 12,500 x *g* for 20 minutes at 4°C. The supernatants were passed through a filter with 40 μm pores and stored at -80°C.

### ELISA and multiplex quantification

IL-1β was quantified with a quantitative ELISA kit, according to the manufacturer’s instructions (Invitrogen, Thermo Fisher). The levels of mouse CCL2, CCL3, CXCL1, CXCL2, EGF, FGF basic, IFNγ, IGF-1, IL-1β, IL-6, IL-10, IL-12p70, IL-13, LIX, MMP-9, oncostatin M, TIMP-1, TNFα and VEGF were determined with a Luminex assay kit (R&D Systems).

### Whole-blood assay and RNA extraction

Twenty-nine patients (14 men and 15 women) diagnosed with Buruli ulcer and treated at the *Centre de Diagnostique et de traitement de la Lèpre et de l’Ulcère de Buruli* CDTLUB were enrolled in this study. We collected 3 mL of whole blood from each subject into a Tempus tube (Applied Biosystem) at diagnosis (Day 0) and at the end of treatment (Day ≥ 56). For subjects remaining at the CDTLUB after the completion of their antibiotic treatment (e.g. for surgery), additional whole-blood samples were collected. Total RNA was extracted with the Tempus Sample kit (Applied Biosystem). In total, 100 blood samples from the 29 patients passed BioAnalyzer (Agilent) quality control, with mean RNA integrity numbers above 8.7 (the lowest value obtained being 6.5), indicating high RNA quality. Transcriptomic data are now available in GEO (GEO number “GSE157350”).

### Analysis of gene expression in blood samples from Buruli ulcer patients

Total RNA was extracted from the whole-blood cells of 29 Buruli ulcer patients at the start and end of antibiotic treatment. The power for identifying genes with true differential expression with a |log2(FC)| > 0.3 was almost 100%, as in previous studies of similar sample size [63]. RNA samples were labeled with the Illumina TotalPrep RNA Amplification Kit from Ambion, hybridized with Illumina HumanHT-12 v4 Expression BeadChips and screened for 47,323 probes. Samples from the same subject were assigned to the same chip for hybridization. Gene expression was assessed with a single microarray per sample. We used the lumi package of R [64] for variance-stabilization transformation (VST) and quantile normalization of the raw data. Only probes corresponding to autosomes, mapping to unique locations and expressed at levels above background noise (*p*-value < 0.05) in at least 10 samples (10%) were retained. Probes not matching any unique Ensembl gene or Hugo ID were excluded. For genes with several probes, we retained the median expression value. Note that probes mapping to regions including known SNPs in the African population of the gnomAD Project (AFR) were not excluded as suggested by Schurmann et al. [64]. We compared the expression of 11,884 genes displaying significant differential expression between the start and end of antibiotic treatment, in paired moderated *t* tests [65] with Benjamini and Hochberg [66] correction for multiple testing.

### Identification of differentially expressed genes and gene ontology enrichment analysis

We used the paired design implemented in the limma package of R software to calculate the log_2_ fold-change (log_2_(FC)) in expression between the start and end of antibiotic treatment (i.e. day 0 vs. ≥ 56 days) for each of the 11,884 genes. *P*-values were obtained for moderated paired *t-*tests [65] and were corrected by the Benjamini-Hochberg procedure [66]. Power calculations with the sizepower package in R software [67] confirmed a power >98% for the detection of a log_2_(FC) > 0.3, as in previous studies of similar sample size [63]. Therefore, only genes displaying a log_2_(FC) ≥ 0.3 were used for the gene ontology enrichment analyses. We used ClueGO Cytoscape module v2.5.4 (GO BiologicalProcess-EBI-UniProt-GOA updated on 2020-08-11) [68] to assess the enrichment in biological processes for the genes differentially expressed between diagnosis and the end of antibiotic treatment (day 0 vs. day ≥ 56). Upregulated genes were tested in a right-sided hypergeometric test, and false discovery rate (FDR) was controlled by the Benjamini-Hochberg method [69]. The Min GO level was set to 3 and the Max GO level to 10. The minimum number of genes per GO term considered was set to 20, and the % Genes per GO term considered was set to 5. The GO Term Grouping function was used, the % of Genes for Group Merge was set at 25%, and visualization as chord diagram was performed with the GOplot package in R [70].

### Statistical analysis

The data, presented as means and SEM (standard errors of mean), are available as raw data in the S1 Data file, and were analyzed with GraphPad Prism 7.0 software (GraphPad Software, San Diego, CA, USA). Within a given cell model, sets of conditions were compared in non-parametric Mann-Whitney *U* tests. ANOVA multiple comparison tests (Dunn’s and Tukey’s tests) were performed for the comparison of several conditions simultaneously. Finally, we controlled for the false discovery rate, by performing Benjamini, Krieger and Yekutieli *t*-tests to compare soluble factors between the tissues of uninfected and infected mice.

Supporting materials and methods

See S1 File.

## Supporting information

S1 Fig Mycolactone does not induce cytotoxic effect, or pro-IL-1β production, at 3 ng/mL. **A.** BMDM and **B.** hMDM cells were stimulated with mycolactone at dose of 0.3, 3 or 30 ng/ml during 48h. Cytotoxic effect was recorded using ToxiLight bioassay kit (Lonza). Dunett’s multiple comparison was realized. **p-value < 0.01, n = 5. **C**. hMDM apoptotic cells in presence of 0, 3 or 30 ng/ml mycolactone was measured with TUNEL Assay, showing a significant increase of apoptosis induced with 30 ng/ml (n = 4 independent human donor, Dunn’s multiple comparison test. *p-value < 0.05). **D.** IL-1β and **E**. IL-6 were detected in supernatant of cells by ELISA. **F.** Pro- IL-1β was detected in cell lysate of cells in timecourse of LPS-stimulation and/or mycolactone incubation. **(**β -Actin was used as control). Materials and methods in the S1 File.
(TIF)

S2 Fig Subcutaneous injection of 12 μg mycolactone induced severe footpad swelling and tissue damages (circle), prevented by dexamethasone. Dexamethasone (10 mg/kg) was administered by oral gavage three days before the subcutaneous injection of mycolactone (12 μg) into the footpad of BALB/c mice. Lesions were photographed 54 h after mycolactone injection.
(TIF)

S3 Fig Detection of IL-12 by immunohistochemistry in active Buruli ulcer lesion. IL-12 was detected by immunohistochemistry in the lesions of patients with active Buruli ulcers (untreated) but not in the lesions of patients with treated Buruli ulcers (antibiotic treatment). Scale bars: 250 μm (inset = 50 μm). Materials and methods in theS1 File.
(TIF)

S4 Fig *M*. *ulcerans* extracellular vesicles (MEVs) were characterized by Nano Tracking Analysis (NTA). **A.** Nanoparticles were illuminated by a laser and their movement under Brownian motion was tracked for 60 s with camera. Representative images are presented. **B.** Five videos (one color per video) were captured to provide significate concentration and size data. Materials and methods in the S1 File.
(TIF)

S1 Table Soluble factors quantified by multiplex ELISA in crushed skin tissues from mice before and after *M*. *ulcerans* subcutaneous inoculation. (XLSX)

S2 Table Genes upregulated at diagnosis compared to treatment completion. (XLSX)

S3 Table Gene ontology enrichment analysis focusing on genes overexpressed at diagnosis compared to treatment completion. (XLSX)

S1 Data Raw data for each figure are available in the supplemental excel file. (XLSX)

S1 File Supporting materials and methods. (DOCX)
