# Supplementary figures and images for: Mycolactone toxin induces an inflammatory response by targeting the IL-1β pathway: Mechanistic insight into Buruli ulcer pathophysiology
Source: PLoS Pathog. 2020 Dec 18;16(12):e1009107. doi: 10.1371/journal.ppat.1009107 (PMC7748131; doi:10.1371/journal.ppat.1009107)

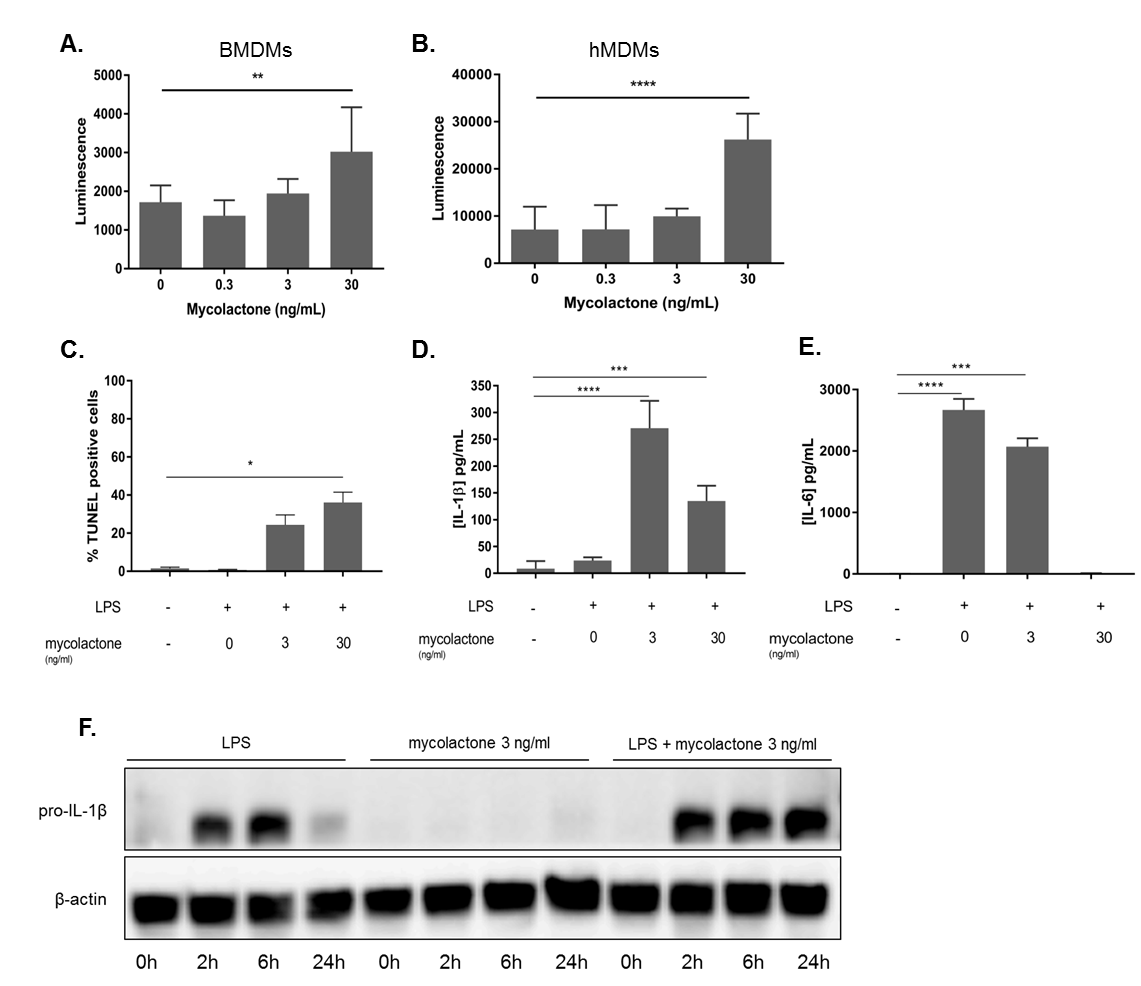

Supplement: S1 Fig — A. BMDM and B. hMDM cells were stimulated with mycolactone at dose of 0.3, 3 or 30 ng/ml during 48h. Cytotoxic effect was recorded using ToxiLight bioassay kit (Lonza). Dunett’s multiple comparison was realized. **p-value < 0.01, n = 5. C. hMDM apoptotic cells in presence of 0, 3 or 30 ng/ml mycolactone was measured with TUNEL Assay, showing a significant increase of apoptosis induced with 30 ng/ml (n = 4 independent human donor, Dunn’s multiple comparison test. *p-value < 0.05). D. IL-1β and E. IL-6 were detected in supernatant of cells by ELISA. F. Pro- IL-1β was detected in cell lysate of cells in timecourse of LPS-stimulation and/or mycolactone incubation. (β -Actin was used as control). Materials and methods in the S1 File. (TIF) [file ppat.1009107.s001.tif]

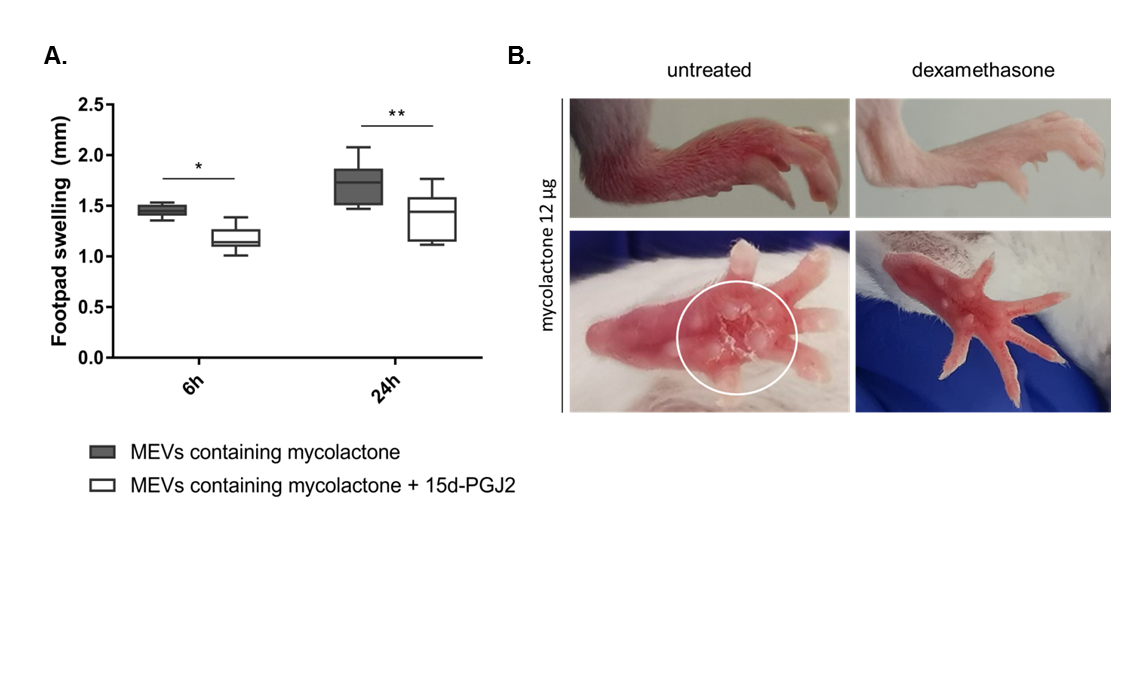

Supplement: S2 Fig — Dexamethasone (10 mg/kg) was administered by oral gavage three days before the subcutaneous injection of mycolactone (12 μg) into the footpad of BALB/c mice. Lesions were photographed 54 h after mycolactone injection. (TIF) [file ppat.1009107.s002.tif]

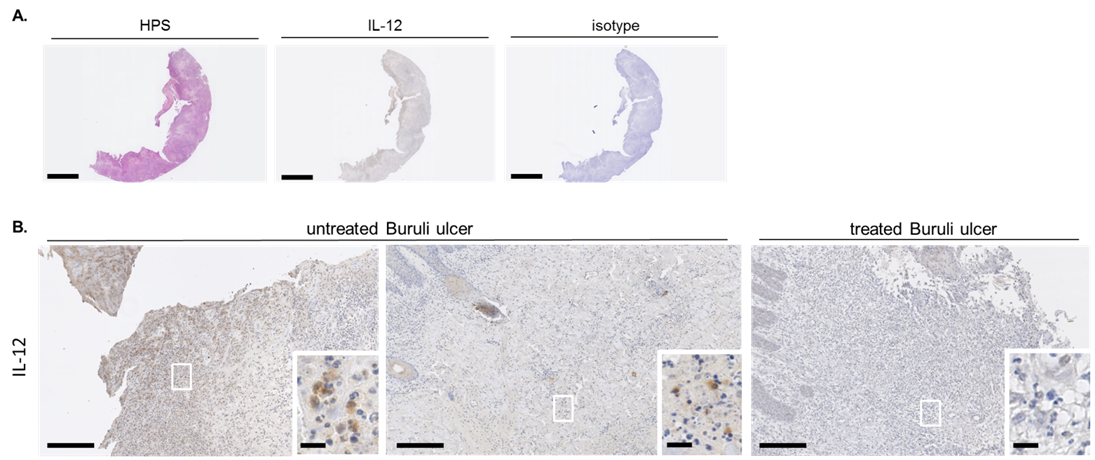

Supplement: S3 Fig — IL-12 was detected by immunohistochemistry in the lesions of patients with active Buruli ulcers (untreated) but not in the lesions of patients with treated Buruli ulcers (antibiotic treatment). Scale bars: 250 μm (inset = 50 μm). Materials and methods in theS1 File. (TIF) [file ppat.1009107.s003.tif]

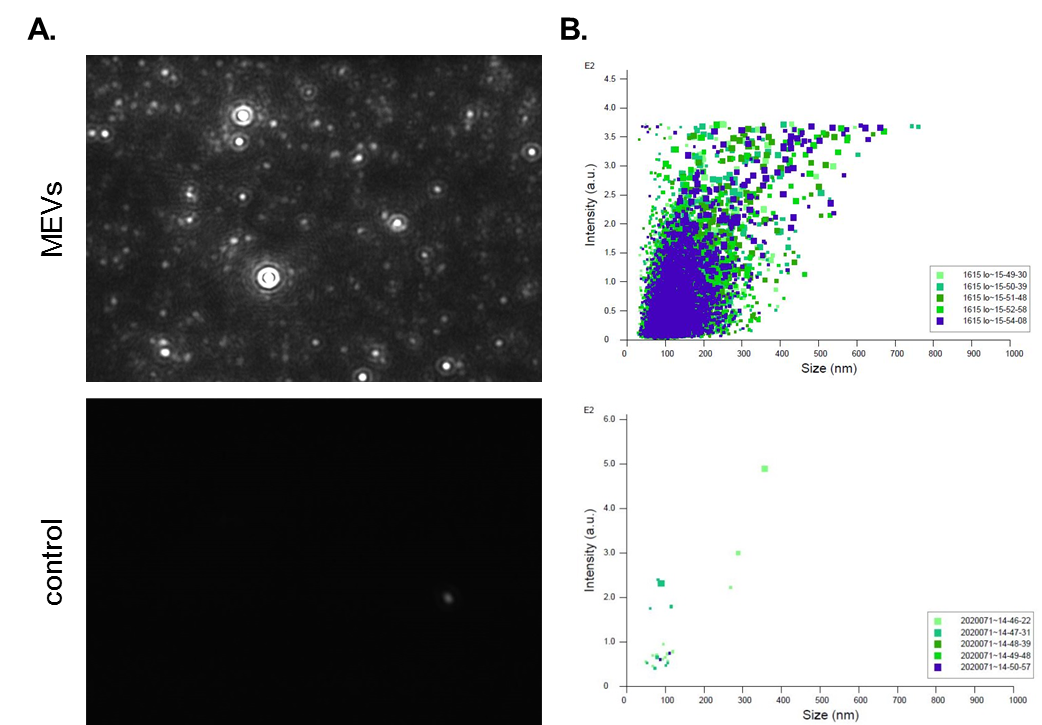

Supplement: S4 Fig — A. Nanoparticles were illuminated by a laser and their movement under Brownian motion was tracked for 60 s with camera. Representative images are presented. B. Five videos (one color per video) were captured to provide significate concentration and size data. Materials and methods in the S1 File. (TIF) [file ppat.1009107.s004.tif]
