# Supplementary material for: Mycolactone toxin induces an inflammatory response by targeting the IL-1β pathway: Mechanistic insight into Buruli ulcer pathophysiology
Source: PLoS Pathog. 2020 Dec 18;16(12):e1009107. doi: 10.1371/journal.ppat.1009107 (PMC7748131; doi:10.1371/journal.ppat.1009107)
Supplement: S1 File — (DOCX) [file ppat.1009107.s009.docx]

**Supplementary materials**

**Cytotoxicity tests**

Toxilight (Lonza) assay were performed on 24h-supernatant of cells incubated in presence of mycolactone at different doses. Briefly, 5 µL of supernatant were transferred to a luminescence compatible 384 well plate (Greiner) and incubated with 25 µL of Adenylate Kinase (AK) detection reagent. Luminescence was measured after 5 min with a luminometer (Biotek Synergy Reader), 5 wells were measured for each condition.

**TUNEL assay**

Apoptosis was measured by using the TUNEL assay kit (Abcam, ref. #ab66110). The principle is based on the detection of DNA fragmentation through terminal deoxynucleotidyl transferase mediated dUTP nick end-labeling. Cells were incubated in defined conditions in up-cell 96-wells microplaques. After 24h, supernatant was recovered and cells were collected (1 x 10^6^ cells) and resuspend in 0.5 mL PBS. Cells were then fixed in 5 mL of 4% paraformaldehyde and incubated 15 min on ice. After three washing steps in PBS, cells were finally resuspended in 5 mL of ice-cold 70% (v/v) ethanol and stored at -20°C for 1 to 2 weeks. For apoptosis labeling, cells were washed three times in wash buffer (kit), then incubated in 50 µL of DNA labeling solution (per test: 10 μL TdT Reaction Buffer, 0.75 μL TdT Enzyme, 8 μL Br-dUTP, 32.25 μL ddH2O) and placed inside a dark humidity incubator at 37 °C for 1 h. After 2 steps of washing in rinse buffer, cells were resuspended in 100 µL of antibody solution (per test: 5 µL Anti-BrdU-Red antibody, 95 µL Rinse Buffer) and incubated in the dark for 30 min at room temperature. 500 µL of 7-AAD/RNase A solution (cell viability marker) were finally added. After incubation 30 min in the dark at room temperature, cells were analyzed by flow cytometry (Ex/Em = 488/576 nm for BrdU-Red and Ex/Em = 488/655 nm for 7-AAD).

**Cell lysates, SDS-PAGE and immunoblotting**

Macrophages were collected after removal of the supernatant and a non-enzymatic treatment with Versene (Gibco) for 5 min. Cells were then resuspended in SDS-lysis buffer containing Laemmli Buffer), and 10 µg (equivalent protein) were subjected to SDS-PAGE on 4-20% precast acrylamide gel (Mini-PROTEAN TGX, Bio-rad) and transferred onto nitrocellulose membranes (Trans-Blot turbo, Bio-rad). The membrane was blocked for 1 hour at room temperature in 5% (w/v) BSA/Tris-buffered saline (TBS) supplemented with 0.1% (v/v) tween-20. Western blot was realized using rabbit anti-IL-1β D3H1Z (Cell Signaling, ref #12507S) diluted at 1:1000, rabbit anti-β-actin (Abcam, ref # ab8227) diluted at 1:10 000, and secondary goat anti-rabbit IgG (H+L) HRP-conjugated (Invitrogen, ref. #G21234) diluted at 1:5000. Detection was performed through application of Pierce ECL substrate (ThermoFisher, ref. #32106).
